# A Rac1 inhibitory peptide suppresses antibody production and paw swelling in the murine collagen-induced arthritis model of rheumatoid arthritis

**DOI:** 10.1186/ar2900

**Published:** 2010-01-06

**Authors:** Joana RF Abreu, Wendy Dontje, Sarah Krausz, Daphne de Launay, Paula B van Hennik, Anne-Marieke van Stalborch, Jean-Paul ten Klooster, Marjolein E Sanders, Kris A Reedquist, Margriet J Vervoordeldonk, Peter L Hordijk, Paul P Tak

**Affiliations:** 1 Division of Clinical Immunology and Rheumatology, Academic Medical Center, University of Amsterdam, Meibergdreef 9, Amsterdam, 1105 AZ, The Netherlands; 2 Department of Molecular Cell Biology, Sanquin Research and Landsteiner Laboratory, Academic Medical Center, University of Amsterdam, Plesmanlaan 125, Amsterdam, 1066 CX, The Netherlands; 3 Arthrogen BV, Meibergdreef 45, Amsterdam, 1105 BA, The Netherlands

## Abstract

**Introduction:**

The Rho family GTPase Rac1 regulates cytoskeletal rearrangements crucial for the recruitment, extravasation and activation of leukocytes at sites of inflammation. Rac1 signaling also promotes the activation and survival of lymphocytes and osteoclasts. Therefore, we assessed the ability of a cell-permeable Rac1 carboxy-terminal inhibitory peptide to modulate disease in mice with collagen-induced arthritis (CIA).

**Methods:**

CIA was induced in DBA/1 mice, and in either early or chronic disease, mice were treated three times per week by intraperitoneal injection with control peptide or Rac1 inhibitory peptide. Effects on disease progression were assessed by measurement of paw swelling. Inflammation and joint destruction were examined by histology and radiology. Serum levels of anti-collagen type II antibodies were measured by enzyme-linked immunosorbent assay. T-cell phenotypes and activation were assessed by fluorescence-activated cell sorting analysis. Results were analyzed using Mann-Whitney *U *and unpaired Student *t *tests.

**Results:**

Treatment of mice with Rac1 inhibitory peptide resulted in a decrease in paw swelling in early disease and to a lesser extent in more chronic arthritis. Of interest, while joint destruction was unaffected by Rac1 inhibitory peptide, anti-collagen type II antibody production was significantly diminished in treated mice, in both early and chronic arthritis. *Ex vivo*, Rac1 inhibitory peptide suppressed T-cell receptor/CD28-dependent production of tumor necrosis factor α, interferon γ and interleukin-17 by T cells from collagen-primed mice, and reduced induction of ICOS and CD154, T-cell costimulatory proteins important for B-cell help.

**Conclusions:**

The data suggest that targeting of Rac1 with the Rac1 carboxy-terminal inhibitory peptide may suppress T-cell activation and autoantibody production in autoimmune disease. Whether this could translate into clinically meaningful improvement remains to be shown.

## Introduction

Rheumatoid arthritis (RA) is marked by de-regulated recruitment, activation, and retention of inflammatory white blood cells in affected joints [1]. Subsequent autoantibody production, release of cytokines, and cell-cell contacts may perpetuate inflammation and lead to joint destruction through activation of stromal fibroblast-like synoviocytes (FLSs) and osteoclasts [2]. Many of the cellular processes required for perpetuation of inflammation and joint destruction in RA are regulated by Rac GTPases, members of the Rho-like family of small GTPase signaling proteins [3].

Rac1 is ubiquitously expressed in mammalian tissues, whereas expression of Rac2 is limited to cells of hematopoietic lineage [4,5]. Rac GTPases are activated by a broad array of extracellular stimuli relevant to RA, including chemokines, lymphocyte antigen receptor ligation, inflammatory cytokines, and cell-cell adhesion [6-11]. Following activation, Rac proteins initiate multiple signaling pathways that regulate cytoskeletal rearrangements, kinase cascades needed for gene transcription, and assembly of the NADPH oxidase [6,12]. Transfection of active and dominant-negative mutants of Rac1 as well as genetic studies have demonstrated that lymphocytes and neutrophils require Rac1 signaling for efficient polarized chemotactic responses and trafficking *in vivo *[13-19]. Although macrophages do not require Rac1 and Rac2 function for chemotactic responses, macrophage invasion of tissue is dependent upon Rac1 and Rac2 [20]. Rac signaling is also important for productive interactions between lymphocytes and antigen-presenting cells (APCs). After antigen recognition by T cells, ezrin-radixin-moesin proteins are dephosphorylated through a Rac1-dependent pathway, favoring relaxation of the cytoskeleton and subsequently promoting T cell-APC conjugate formation [21]. Reciprocally, Rac activity in dendritic cells (DCs) is required for effective antigen presentation to T cells and subsequent T-cell priming [22]. Antigen receptor-dependent activation of Rac signaling also stimulates activation of mitogen-activated protein kinase, phosphatidylinositol 3-kinase, and nuclear factor-kappa-B signaling pathways important for lymphocyte activation, proliferation, and survival [7-9]. Many of these downstream signaling pathways are now being explored as potential therapeutic targets in RA [23]. Rac proteins also serve additional important functions in cells of myeloid lineage which contribute to inflammation and joint destruction in RA. Oxidative bursts of macrophages and neutrophils rely upon Rac1-dependent assembly of the NADPH oxidase machinery [12]. Additionally, *in vitro *studies of osteoclasts transfected with plasmid encoding dominant-negative Rac and *in vivo *studies in Rac-deficient mice have identified essential but redundant roles for Rac1 and Rac2 proteins in osteoclastogenesis, osteoclast motility, and bone resorption [24,25].

Together, these studies indicate that therapeutic strategies targeting Rac1 function may be of clinical benefit in RA. However, preclinical assessment of Rac1 inhibition has been hampered by a lack of compounds specifically targeting Rac1 and by limited analyses of Rac1 in animal models of arthritis, a consequence of early findings demonstrating that genetic deletion of Rac1 in mice results in early embryonic lethality [26]. NSC23766, a pharmacological compound that inhibits Rac GTPases via targeting of the activating guanine nucleotide exchange factors Tiam1 and Trio, suppresses RA FLS proliferation and invasiveness *in vitro*, effects mimicked by siRNA (short interfering RNA) silencing of Rac1 expression in these cells [27]. This may indicate that specific inhibition of Rac1 may be therapeutically beneficial in RA. However, mice in which Rac1 has been conditionally deleted in mature neutrophils and macrophages on a Rac2-deficient background show a complex phenotype in a *Chlamydia*-induced infection model of arthritis [28]. In these animals, Rac1 has a bimodal effect on disease progression. In the acute phase, Rac1 deficiency delays recruitment and activation of inflammatory neutrophils in the joint, whereas in the chronic phase, disease is exacerbated due to an inability of neutrophils to clear the pathogen. In this study, we targeted Rac1 in mice with collagen-induced arthritis (CIA), using a Rac1-specific cell-permeable carboxy-terminal inhibitory peptide that we have previously shown to block Rac1 function in human lymphocytes, endothelial cells, and epithelial cells [11,29,30].

## Materials and methods

### Animals

Male DBA/1 mice were purchased from Harlan (Horst, The Netherlands), housed under conventional conditions at the animal facility of the Academic Medical Center (Amsterdam, The Netherlands), and fed *ad libitum*. The animal ethical committee of the Academic Medical Center approved all experiments.

### Peptide synthesis

For this study, peptides encoding a protein transduction domain [31] alone (indicated as control, Ctrl, throughout the article) or fused to the carboxy-terminal domain of Rac1, excluding the Rac1 CAA*X *box (indicated as Rac1 throughout this article), were synthesized using *N*-(9-fluorenyl)methoxycarbonyl (fMoc) solid-phase chemistry [29]. Peptide synthesis was performed using a Syro II (MultiSynTech GmbH, Witten, Germany).

### T-cell isolation

Murine spleens were crushed through a 40-μm cell strainer (BD Biosciences Pharmingen, San Diego, CA, USA) to obtain single-cell suspensions. Erythrocytes were lysed with ice-cold isotonic NH_4_Cl solution (155 mM NH_4_Cl, 10 mM KHCO_3_, and 100 mM ethylenediaminetetraacetic acid [EDTA], pH 7.4). To purify T cells, splenic cell suspensions were incubated with anti-murine CD4 and CD8 antibody-coated magnetic microbeads (Miltenyi Biotec, Bergisch Gladbach, Germany) for 20 minutes at 4°C and positively selected by magnetic separation with magnetic-activated cell sorting. Purified T cells were more than 90% CD3^+ ^as analyzed by flow cytometry.

### Actin polymerization assays

Actin polymerization assays were performed as previously described [32]. Briefly, purified T cells were preincubated for 15 minutes in medium containing 200 μg/mL Ctrl or Rac1 peptide. T cells were then exposed to 100 ng/mL stromal cell-derived factor 1 alpha (SDF-1α), and at the indicated time points, 100-μL aliquots of cell suspensions were transferred to an equivalent volume of fixation solution (Intraprep Fixation Reagent; Coulter Immunotech, Marseille, France). After 15 minutes, cells were washed in 0.5% bovine serum albumin/phosphate-buffered saline (PBS) and resuspended in 100 μL of permeabilization reagent (Coulter Immunotech) for 5 minutes. Cells were stained for 20 minutes with 1 unit/mL Alexa 488 Phalloidin (Molecular Probes Inc., now part of Invitrogen Corporation, Carlsbad, CA, USA) to visualize F-actin. The mean fluorescence intensity (MFI) of polymerized actin was measured by flow cytometry (BD Biosciences Pharmingen), and the fold increase in actin polymerization was calculated by dividing the MFI generated at a particular time point by the MFI at *t *= 0 of that particular condition.

### Induction and assessment of CIA

Bovine collagen type II (bCII) (2 mg/mL in 0.05 M acetic acid; Chondrex, Inc., Redmond, WA, USA) was mixed with complete Freund's adjuvant (CFA) (2 mg/mL of *Mycobacterium tuberculosis*; Chondrex, Inc.) and injected intradermally on day 0 at the base of the tail with 100 μL of emulsion into 8- to 11-week-old mice. On day 21, mice received an intraperitoneal booster injection with 100 μg of bCII in PBS. To investigate the treatment efficacy of Rac1 peptide at disease onset, mice were treated at day 20 with 2 mg, 1 mg, or 0.5 mg of Ctrl or Rac1 peptide three times weekly until sacrifice. To assess the effects of Ctrl and Rac1 peptides on lymphocyte trafficking, phenotype, and activation, a separate experiment in which 16 mice were injected with bCII in CFA at day 0 as above was performed. On day 19, the mice were randomly assigned, and six mice were sacrificed to obtain splenocytes. The remaining mice were treated with 2 mg of Ctrl or Rac1 peptide (n = 5 for each group) starting from day 20 and were boosted with bCII on day 21, followed by additional treatment with peptide on days 22, 25, and 27. On day 28, mice were sacrificed and blood and spleens were harvested. Alternatively, to explore the effect of Rac1 peptide treatment in chronic disease, the animals were randomly assigned at day 29 to one of two groups and treated intraperitoneally with 4 mg of Ctrl or Rac1 peptide. Treatments were continued three times weekly until sacrifice at day 39. The severity of arthritis was assessed in a blinded manner using a semiquantitative scoring system (0 to 4): 0, normal; 1, redness or swelling or both in one joint; 2, redness or swelling or both in more than one joint; 3, redness or swelling or both in the entire paw; and 4, deformity or ankylosis or both. Hind paw ankle joint thickness was measured using a dial caliper (POCO 2T 0- to 10-mm test gauge; Kroeplin Längenmesstechnik, Schlüchtern, Germany). Experiments were performed using 8 to 16 mice per group.

### Histological analysis

Hind paws were fixed in 10% buffered formalin for 48 hours and decalcified in 15% EDTA. The paws were then embedded in paraffin, and 5-μm saggital serial sections of whole hind paws were cut. Tissue sections were stained with hematoxylin and eosin. Inflammation was graded on a scale from 0 (no inflammation) to 3 (severely inflamed joint) based on infiltration of the synovium by inflammatory cells. Cartilage erosion was scored using a semiquantitative scoring system from 0 (no erosions) to 3 (extended erosions). The tissue was examined by microscopic evaluation in a blinded manner by two independent observers (JRFA and MJV).

### Radiological analysis

Hind paws were used for radiographic evaluation. Two observers without knowledge of the treatment groups scored the x-rays. Joint destruction was scored on a scale from 0 to 4: 0, no damage; 1, minor bone destruction observed in one enlightened spot; 2, moderate changes, two to four spots in one area; 3, severe erosions afflicting the joint; and 4, complete destruction of the joints.

### Determination of anti-collagen antibodies by enzyme-linked immunosorbent assay

Maxisorb 96-well plates (Nunc, Roskilde, Denmark) were coated with 5 μg/mL of bCII in 0.1 M sodium carbonate buffer (pH 9.7) overnight at 4°C. After blocking for 1 hour with 2% milk in PBS at room temperature, sera were added in serial dilutions in 2% milk/PBS and incubated overnight at 4°C. Plates were subsequently washed and incubated with 1 μg/mL biotinylated rat anti-mouse immunoglobulin (Ig) (SouthernBiotech, Birmingham, AL, USA) of the indicated isotype in 2% milk/PBS for 1 hour at room temperature. After washing, plates were incubated with streptavidin-conjugated alkaline phosphatase (Stratech Scientific Limited, Newmarket, Suffolk, UK) for 1 hour at room temperature, washed, and developed with p-nitrophenyl phosphate substrate (Sigma-Aldrich, St Louis, MO, USA). The reaction was stopped with 2 M H_2_SO_4_, and optical density at 415 nm was measured.

### B- and T-cell phenotyping

Splenocytes were obtained as above. White blood cells were obtained from approximately 100 μL of blood following heart puncture. Erythrocytes were removed by lysis in isotonic NH_4_Cl solution and the remaining white blood cells were counted prior to staining. Antibodies from eBioscience, Inc. (San Diego, CA, USA) used in this study included the following: anti-CD3-fluorescein isothiocyanate (FITC), -APC; anti-B220-FITC, -APC; anti-CD8-phycoerythrin (PE), -FITC, -Alexa 780; anti-CD44-FITC, -PerCP Cy5.5; anti-CD62L-APC, -PeCY7; anti-Foxp3-PE, -APC; anti-interleukin (IL)-17-Alexa 488; anti-tumor necrosis factor alpha (TNFα)-APC, -PE; and anti-ICOS-PE and anti-CD154-APC. Anti-CD4-FITC, anti-CD4-PE, and anti-IL-2-APC were from BD Biosciences Pharmingen. For phenotyping, splenocytes and white blood cells were stained with the indicated fluorochrome-conjugated antibodies. To assess T-cell cytokine expression, splenocytes obtained from mice on day 19 following immunization with bCII in CFA immunization were pretreated for 15 minutes with 200 μg/mL Ctrl and Rac1 peptides, followed by? stimulation for 24 hours with plate-bound anti-CD3 (5 μg/mL) and soluble anti-CD28 (5 μg/mL) antibodies (both generously provided by Dr Louis Boon, Bioceros BV, Utrecht, The Netherlands). During the last 4 hours of stimulation, brefeldin A (10 μg/mL; Sigma-Aldrich) was added to the splenocyte cultures. Cells were harvested and stained with CD3, CD4, and CD8 antibodies, followed by fixation, permeabilization with Cytofix/Cytoperm (BD Biosciences Pharmingen), and labeling for intracellular cytokines. Treatment of cells with brefeldin A and permeabilization were omitted in assessment of ICOS and CD154 expression. Cell marker expression and cytokine expression were detected using a FACS [fluorescence-activated cell sorting] Calibur flow cytometer (BD Biosciences Pharmingen) and CellQuest Pro software (BD Biosciences Pharmingen).

### Statistical analysis

To evaluate the effects of different treatments, we determined the change in paw swelling scores (delta) of each mouse from the start of treatment until the end of the experiment. Areas under the curve were calculated for the delta paw swelling. The significance of the differences in delta paw swelling, radiological, and histological scores between groups was determined using the unpaired Student *t *test. Potential differences in T- and B-lymphocyte surface marker expression and cytokine production were determined using a Mann-Whitney *U *test. *P *values of not more than 0.05 were considered statistically significant.

## Results

### The Rac1 inhibitory peptide blocks murine T-cell actin polymerization

**Figure 1:**
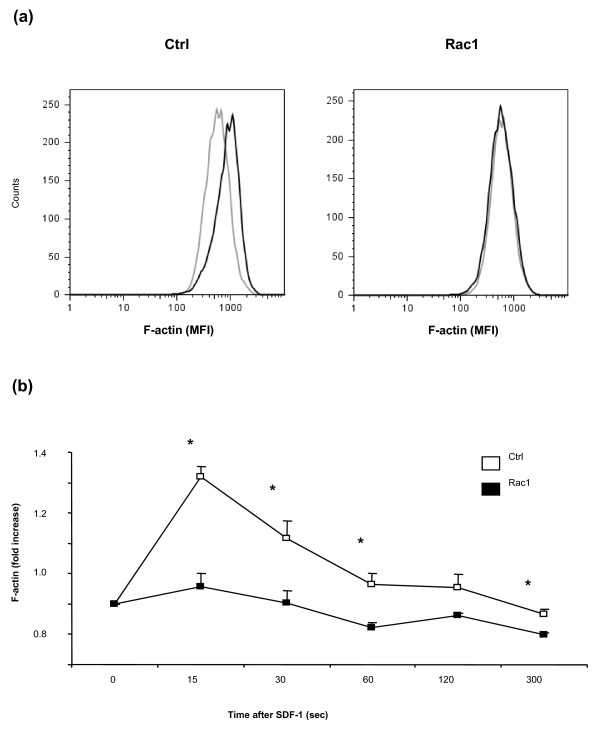
**The Rac1 carboxy-terminal peptide efficiently blocks actin polymerization in murine cells**. **(a) **Representative histograms of F-actin staining in T cells isolated from mice spleens that were exposed for 15 minutes to 200 μg/mL of Ctrl or Rac1 peptide followed by stromal cell-derived factor 1 (SDF-1) stimulation. **(b) **Fold increase in F-actin. Data are depicted as mean fold increase (MFI) of F-actin ± standard error of the mean (n = 3). *P < 0.05. Ctrl, control.

The Rac1 peptide is able to block endogenous Rac1 signaling within minutes by competing with Rac1 effector proteins and, *in vitro*, has been demonstrated to possess a potent capacity to block actin polymerization and migration of human cells stimulated with SDF-1α [11,29,30]. The biological activity of the peptide batches used for *in vivo *experiments and their ability to influence murine cellular responses were first examined in an actin polymerization assay using murine splenic T cells. Pretreatment of murine T cells with Rac1 peptide, but not Ctrl peptide, completely blocked actin polymerization following SDF-1α stimulation, as measured by increases in T-cell F-actin content (Figure 1a, b). This indicated that the Rac1 peptide was effective in blocking Rac1 signaling not only in human cells but in murine cells as well.

### Rac1 inhibitory peptide treatment reduces paw swelling and anti-bCII antibody production in early arthritis

**Figure 2:**
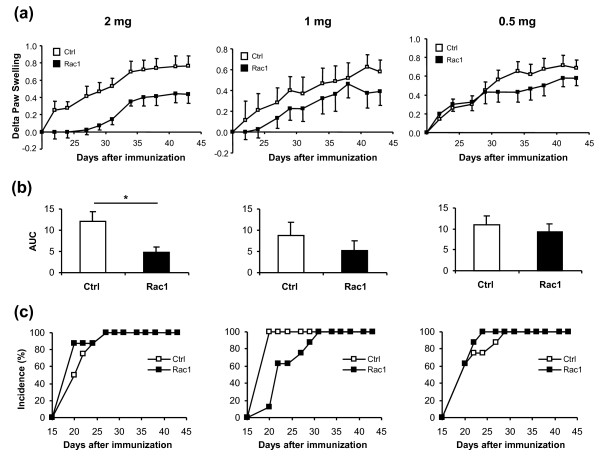
**Reduced paw swelling, but no effect on disease incidence, after treatment with 2 mg of Rac1 carboxy-terminal peptide**. Mice were treated with 2 mg, 1 mg, or 0.5 mg of Ctrl (white square) or Rac1 (black square) peptide at the indicated time points. Paw swelling and inflammation of the four limbs were determined for each mouse. **(a) **Delta hind paw ankle joint swelling was calculated by subtracting the paw diameter on the day of initiation of treatment from the measured diameter. Values are presented as mean ± standard error of the mean (SEM) (n = 8). **(b) **Area under the curve (AUC) was calculated for the delta paw swelling in each mouse. Values are presented as mean AUC ± SEM. *P < 0.05. **(c) **Cumulative incidence of arthritis in mice for each dosage of Ctrl and Rac1 peptide was calculated. Ctrl, control.

After confirming the *in vitro *efficiency of the Rac1 peptide in inhibiting murine Rac1 signaling, we examined the *in vivo *potential of this peptide when mice are treated at the onset of disease. One day before the booster at day 20, we started treatment of the animals with 2 mg, 1 mg, or 0.5 mg of Ctrl or Rac1 peptide three times weekly until sacrifice. Treatment of animals with Rac1 peptide at all doses failed to influence clinical scores of disease severity or animal weight (data not shown), but animals treated with 2 mg of Rac1 peptide showed a highly significant decrease in paw swelling when compared with treatment with Ctrl peptide (61% reduction, *P *= 0.009) (Figure 2a, b). The effect was dose-dependent; treatment with lower doses of Rac1 peptide showed a trend toward improvement, and this trend did not reach statistical significance (Figure 2a, b). Administration of Rac1 peptide had no significant effects on disease incidence or onset in these experiments (Figure 2c).

**Figure 3:**
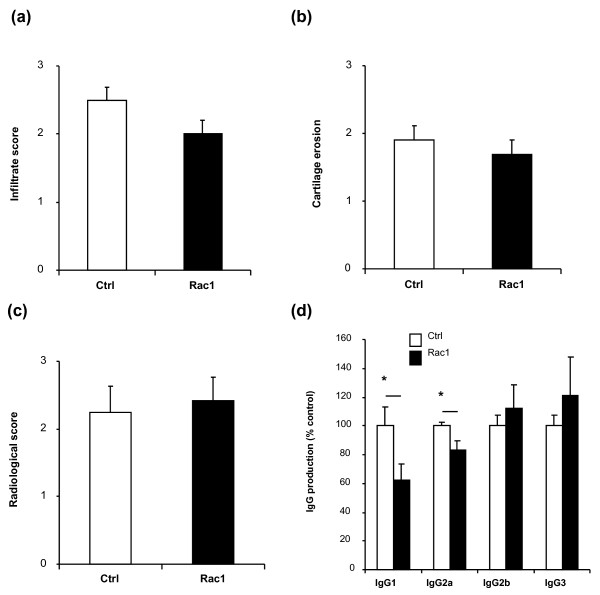
**Effects of treatment with the Rac1 carboxy-terminal peptide at onset of disease in collagen-induced arthritis**. Mice were treated with 2 mg of Ctrl (white bars) or 2 mg of Rac1 (black bars) peptide starting at day 20. After sacrifice, sections from mice paws (n = 8 per group) were stained with hematoxylin and eosin and assessed for **(a) **cellular infiltration and **(b) **cartilage erosion. **(c) **X-rays of hind paws were analyzed for bone damage. **(d) **Sera from mice that started treatment at day 20 (n = 8) with Ctrl (white bars) or Rac1 (black bars) peptide were collected, and the levels of specific anti-collagen IgG were detected. IgG levels in the sera of Ctrl-treated mice were set to 100%, and the levels obtained in the sera of Rac1-treated mice were then calculated relative to Ctrl. Represented IgG values were calculated within linear regions of the serum dilution curve. All values are presented as mean ± standard error of the mean. *P ≤ 0.05. Ctrl, control.

We further examined mice treated with 2 mg of Ctrl and Rac1 peptides for other disease parameters. Quantification of synovial inflammation revealed a minor decrease in cellularity, but this decrease did not reach statistical significance (Figure 3a). Treatment with Rac1 peptide did not protect against joint destruction (Figure 3b, c). Finally, we examined the influence of Rac1 peptide on anti-bCII antibody production. We collected sera from mice at the time of sacrifice and measured specific anti-bCII antibody levels by enzyme-linked immunosorbent assay. Mice treated with 2 mg of Rac1 peptide showed a significant reduction in the serum levels of anti-bCII IgG1 (Ctrl 100% ± 12.9%; Rac1 62.1% ± 11.8%; *P *< 0.05) and IgG2a (Ctrl 100% ± 2.7%; Rac1 83.3% ± 6.8%; *P *= 0.05) antibodies compared with mice treated with Ctrl peptide (Figure 3d).

### Treatment of chronic CIA with Rac1 peptide reduces anti-bCII antibody production

**Figure 4:**
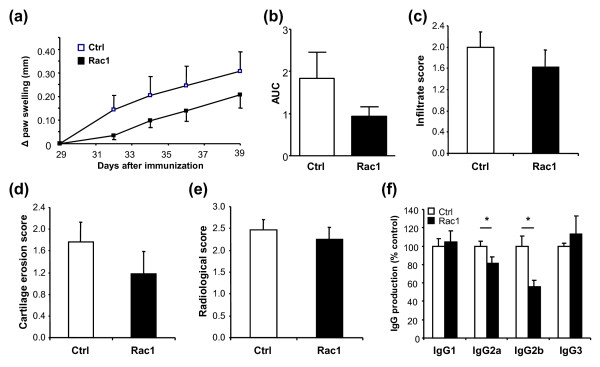
**Effect of Rac1 carboxy-terminal peptide treatment of mice with chronic arthritis**. **(a) **Delta hind paw ankle joint swelling of mice that started treatment at day 29 with 4 mg of Ctrl or Rac1 peptide (three times weekly). Values are representative of two independent experiments (n = 16 per group). **(b) **Area under the curve (AUC) calculated as in Figure 2b. Paraffin-embedded sections of the hind paws were stained with hematoxylin and eosin and analyzed for **(c) **synovial inflammation and **(d) **cartilage destruction. **(e) **X-rays were analyzed for bone damage (n = 16 per group). **(f) **Sera from mice that started treatment at day 29 (n = 7) with Ctrl (white bars) or Rac1 (black bars) peptide were collected, and the levels of specific anti-collagen IgG were detected. IgG levels in the sera of Ctrl-treated mice were set to 100%, and the levels obtained in the sera of Rac1 peptide-treated mice were then calculated relative to Ctrl. Represented values were calculated within linear regions of the serum dilution curve. All values are expressed as mean ± standard error of the mean. *P ≤ 0.05. Ctrl, control.

We next investigated the effect of Rac1 peptide treatment on mice with chronic arthritis. For this, we performed an independent CIA experiment wherein 29 days after the initial immunization, mice having clinical signs of arthritis were randomly assigned to one of two groups. Mice within groups were treated with 4 mg of Ctrl or 4 mg of Rac1 peptide three times weekly until sacrifice. Administration of Rac1 peptide had no influence on clinical disease severity (data not shown). However, we observed a clear trend toward reduced paw swelling in mice treated with Rac1 peptide (Figure 4a, b) in two independent experiments, although the differences did not reach statistical significance (experiment 1: 49% reduction, *P *= 0.528; experiment 2: 22% reduction, *P *= 0.193) (Figure 4b).

We also analyzed the effects of the Rac1 peptide on synovial inflammation, cartilage degradation, and bone destruction in this model. Consistent with the trend toward reduced paw swelling, there was a trend toward decreased histological signs of inflammation and cartilage destruction, but this trend did not reach statistical significance (Figure 4c, d). Analysis of x-rays taken from the mice paws revealed that Rac1 peptide treatment did not protect against erosive disease (Figure 4e). However, Rac1 peptide treatment of mice with chronic arthritis resulted in a significant reduction in the serum levels of anti-bCII IgG2a (Ctrl 100% ± 5.6%; Rac1 81.6% ± 6.5%; *P *= 0.05) and IgG2b (Ctrl 100% ± 10.9%; Rac1 56.0% ± 6.7%; *P *< 0.005), whereas no differences were observed for IgG1 or IgG3 (Figure 4f).

### Rac1 peptide does not alter the distribution or phenotype of lymphocytes during the onset of arthritis

**Figure 5:**
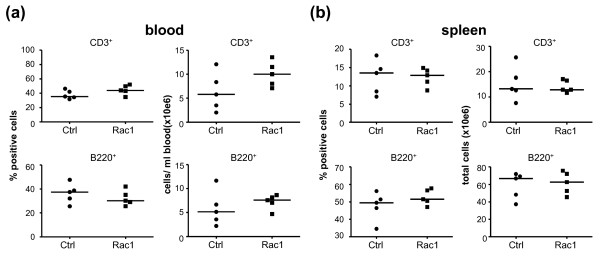
**Effect of Rac1 carboxy-terminal peptide treatment of mice on lymphocyte numbers and percentages in blood and spleen**. Mice treated with Ctrl or Rac1 peptide (2 mg, n = 5 per group) were sacrificed on day 28 following arthritis induction, and CD3^+ ^T cells and B220^+ ^B cells were identified by fluorescence-activated cell sorting analysis in **(a) **blood and **(b) **spleen. Numbers of CD3^+ ^T cells (upper panels) and B220^+ ^cells (lower panels) were calculated as percentage positive cells (left panels) or total number of cells (right panels) per milliliter of blood or per spleen. Data points within each column represent individual mice, and the bar indicates the median value. Ctrl, control.

**Figure 6:**
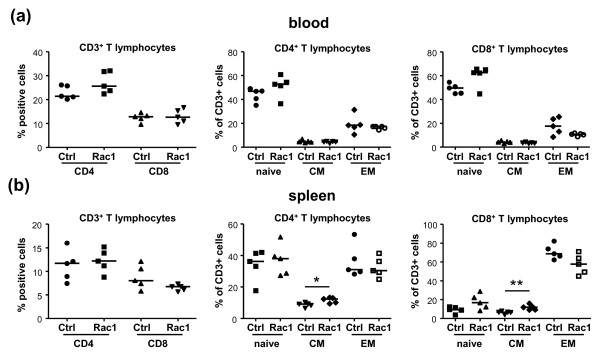
**T-cell phenotyping in arthritic mice treated with Rac1 carboxy-terminal peptide**. Blood **(a) **and spleens **(b) **were obtained from arthritic mice treated with Ctrl or Rac1 peptide (2 mg, n = 5 per group), and cells were stained with antibodies against CD3, CD4, CD8, CD44, and CD62L. The percentages of CD3^+ ^T cells expressing CD4 and CD8 (left panels) and naïve (CD44^-^CD62L^+^), central memory (CM) (CD44^+^CD62L^+^), and effector/memory (EM) (CD44^+^CD62L^-^) CD3^+^CD4^+ ^(middle panels) and CD3^+^CD8^+ ^T cells were calculated. Data points within each column represent individual mice, and the bar indicates the median value. *P ≤ 0.05, *P ≤ 0.01. Ctrl, control.

To better understand how Rac1 peptide might influence paw swelling and autoantibody formation during the onset of CIA, we examined the distribution of B and T lymphocytes in mice shortly after disease onset. Mice were immunized with bCII in CFA, and one day before administrating a second immunization with bCII on day 20, we randomly assigned the mice and initiated treatment with 2 mg of Ctrl or Rac1 peptide (n = 5 mice per group). Mice were treated with peptides three times weekly until sacrifice on day 28. Total numbers and percentages of B and T lymphocytes in blood (Figure 5a) and spleens (Figure 5b) were then assessed. As compared with mice treated with Ctrl peptide, Rac1 peptide exerted no effect on either the percentages (left panels) or total numbers (right panels) of CD3^+ ^T lymphocytes (upper panels) or B220^+ ^B lymphocytes (lower panels). We also addressed the possibility that Rac1 peptide may regulate the distribution or differentiation of distinct T-cell subsets during the onset of CIA (Figure 6). However, we observed no differences between Ctrl peptide- and Rac1 peptide-treated mice in regard to CD4^+ ^and CD8^+ ^percentages in blood (Figure 6a, left panel) or in terms of the distribution of CD4^+ ^(middle panel) or CD8^+ ^(right panel) CD44^-^CD62L^+ ^naïve, CD44^+^CD62L^- ^effector/memory, or CD44^+^CD62L^+ ^central memory T cells. Small increases in the percentages of CD4^+ ^(*P *< 0.05) and CD8^+ ^(*P *< 0.01) central memory T cells were observed in the spleens of mice treated with Rac1 peptide, but reciprocal decreases in other T-cell subsets were not evident (Figure 6b). No differences in the percentages of splenic FoxP3^+ ^regulatory T cells were observed (data not shown).

### Rac1 peptide suppresses T-cell receptor-dependent inflammatory cytokine production and T-cell costimulatory protein expression

**Figure 7:**
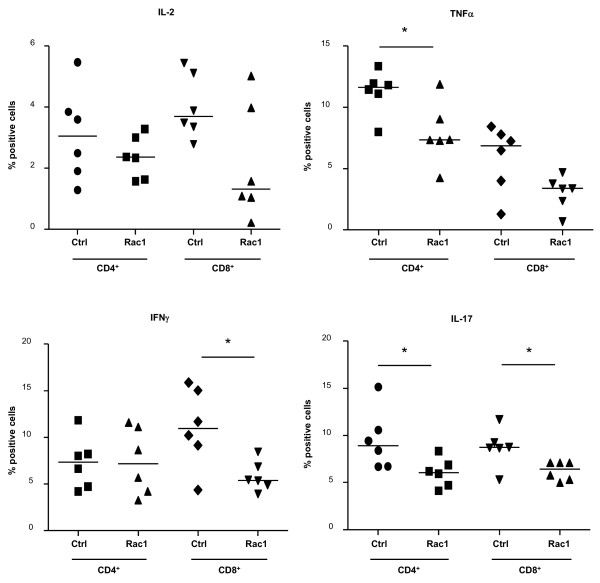
**Effect of Rac1 carboxy-terminal peptide treatment on T-cell cytokine production**. Splenocytes obtained from mice (n = 6) on day 19 following priming with bovine collagen type II in complete Freund's adjuvant were preincubated for 15 minutes with Ctrl or Rac1 peptide (200 μg/mL) and stimulated for 24 hours with anti-CD3 and anti-CD28 antibodies. Brefeldin A was included for the last 4 hours of stimulation, and cells were stained for CD3, CD4, CD8, interleukin (IL)-2, tumor necrosis factor-alpha (TNFα), interferon-gamma (IFNγ), and IL-17. The percentages of CD3^+^CD4^+ ^and CD3^+^CD8^+ ^T cells expressing each cytokine were determined by fluorescence-activated cell sorting analysis. Data points within each column represent values obtained from splenocytes of individual mice, and the bar indicates the median value. *P ≤ 0.05. Ctrl, control.

**Figure 8:**
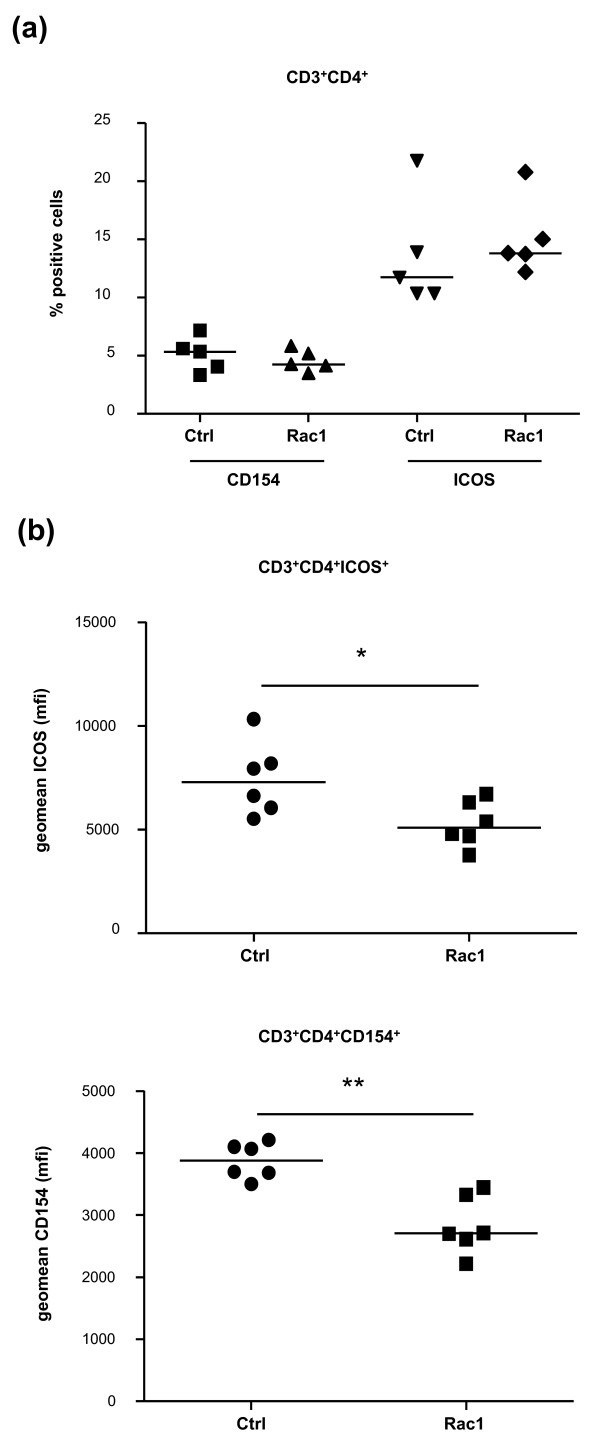
**Effect of Rac1 carboxy-terminal peptide treatment on T-cell costimulatory protein expression**. Splenocytes from mice primed with bovine collagen type II in complete Freund's adjuvant analyzed in Figure 7 (n = 6) were examined for ICOS and CD154 expression by fluorescence-activated cell sorting analysis following 24-hour stimulation with anti-CD3 and anti-CD28 antibodies in the presence of Ctrl or Rac1 peptide (200 μg/mL). The percentages of CD3^+^CD4^+ ^T cells expressing CD154 and ICOS **(a) **and the mean geometric (geomean) fluorescence intensity (mfi) of CD154 and ICOS expression on these cells **(b) **were calculated following fluorescence-activated cell sorting analysis. Data points within each column represent values obtained from splenocytes of individual mice, and the bar indicates the median value. *P ≤ 0.05; **P < 0.01. Ctrl, control.

Our analysis of B- and T-lymphocyte distribution in mice during CIA onset failed to reveal a prominent effect of Rac1 peptide on lymphocyte trafficking, and synovial cellular infiltration scores were similar in mice treated with Ctrl and Rac1 peptides (Figure 3a). We therefore examined whether the effect of the Rac1 peptide on autoantibody production might reflect effects on T-cell activation. Mice (n = 5) were immunized with bCII and CFA and sacrificed on day 19. Splenocytes isolated from the mice were then stimulated with anti-CD3/CD28 antibodies in the presence of Ctrl or Rac1 peptide. Intracellular staining and FACS analysis revealed no effect of Rac1 peptide on T-cell IL-2 production (Figure 7). However, Rac1 peptide significantly decreased the percentages of T cells capable of responding to CD3/CD28 stimulation by production of other T-cell cytokines important in CIA and RA. Significant reductions in the percentages of CD4^+ ^T cells producing TNFα (*P *< 0.05), interferon-gamma (IFNγ)-producing CD8^+ ^T cells (*P *< 0.05), and IL-17-producing CD4^+ ^and CD8^+ ^T cells (*P *< 0.05) were observed. We also examined the effect of the Rac1 peptide on the induced expression of T-cell costimulatory proteins, ICOS and CD154, which play crucial roles in CD4^+ ^T-cell help to B cells needed for autoantibody production in CIA [33-36]. Although Rac1 peptide had no influence on the percentages of CD3^+^CD4^+ ^splenocytes expressing ICOS and CD154 following anti-CD3/CD28 stimulation (Figure 8a), significant reductions in the amount of ICOS (*P *< 0.05) and CD154 (*P *< 0.01) expressed on the T-cell surface were observed following Rac1 peptide treatment (Figure 8b). Thus, interference with T-cell Rac1 signaling directly interferes with the ability of T cells to produce inflammatory cytokines and express costimulatory proteins required for the promotion of autoantibody responses.

## Discussion

In this report, we provide evidence that the inhibitory Rac1 carboxy-terminal peptide suppresses anti-collagen antibody production associated with reduced paw swelling in mice with CIA. We have found that the administration of Rac1 peptide *in vivo*, either as an early treatment or as treatment of mice with more chronic arthritis, results in a significant reduction of circulating levels of anti-bCII IgG1 and IgG2a or IgG2a and IgG2b antibodies, respectively, depending on the stage of the disease. In both early and chronic arthritis, there was an effect on IgG2a levels. In many murine *in vivo *model systems, IgG2a and IgG2b antibodies display greater pro-inflammatory properties than IgG1 and IgG3 [37]. Initial evidence presented here indicates that Rac1 inhibitory peptide exerts its protective effect at least in part through interference with T-cell receptor (TCR)/CD28 signals needed for T-cell expression of inflammatory cytokines as well as for expression of costimulatory proteins required for B-cell activation.

It is unlikely that the suppressive effect of Rac1 peptide on paw swelling and anti-collagen antibody production observed in our studies is due to inhibitory effects on immune cell trafficking. First, we noted no significant differences in cellular infiltration of the synovium in Ctrl and Rac1 peptide-treated mice. Second, the Rac1 peptide did not influence the numbers or percentages of B and T lymphocytes in blood or spleen during CIA. These results are consistent with genetic studies indicating that Rac1 and Rac2 play redundant roles in lymphocyte and myeloid cell trafficking, reflecting the high specificity of the Rac1 peptide for interfering with signaling from Rac1 but not other Rho family GTPases [29]. Murine neutrophils express both Rac1 and Rac2, and genetic deletion of each GTPase has revealed their important but redundant contributions to neutrophil chemotactic responses *in vitro *and *in vivo *[18,19]. Rac1-deficient murine neutrophils retain chemokinetic responses but are unable to orient and migrate toward chemokine gradients. In contrast, Rac2 is required for efficient neutrophil migration [19]. Although similar direct comparative analyses have not been performed on B and T cells in these mice, initial studies indicate that lymphocyte trafficking is regulated primarily by Rac2 [15].

Surprisingly, we observed little if any effect of Rac1 peptide on cartilage and joint destruction in murine arthritis, although a significant decrease in paw swelling was observed in mice when treated at the onset of disease, and a reproducible trend toward reduced paw swelling was noted in mice treated with Rac1 peptide at a more chronic phase of disease. Decreases in paw swelling after treatment with Rac1 peptide which we observed might be due to effects on the formation of edema. Experiments conducted *in vitro *have demonstrated that exposure of human endothelial cells to reactive oxygen species or engagement of the integrin ligand VCAM (vascular cell adhesion molecule) leads to Rac1-dependent loss of cadherin-mediated endothelial cell-cell adhesion. In the presence of the Rac1 peptide, endothelial cell-cell adhesion is maintained (data not shown) [11,38]. A role for Rac1 in maintaining vascular endothelial integrity *in vivo *is also indirectly suggested in studies of c-Jun knockout mice, in which inhibition of c-Jun, a downstream target of Rac signaling, suppresses edema, paw swelling, and inflammation in an experimental model of arthritis [39]. Lack of effect of Rac1 peptide on joint destruction in CIA may reflect a redundant role for Rac2 in supporting osteoclastogenesis [24,25].

Rac1 inhibitory peptide may inhibit anti-bCII antibody production in CIA via one or more mutually non-exclusive mechanisms. It is unlikely that Rac1 inhibition directly interferes with B-cell activation as Rac1 and Rac2 make critically redundant contributions to the transduction of B-cell receptor signals required for both survival and efficient cell cycle entry [9,40]. Rac2 deletion in mice results in decreased B-cell maturation and T cell-independent antigen responses [14,15]. In contrast, conditional deletion of Rac1 in the B-cell compartment has no observable effect on B-cell maturation or function unless Rac2 is simultaneously deleted [40]. Similarly redundant but critical roles for Rac1 and Rac2 are observed during T-cell maturation and proliferative responses to TCR triggering [17]. Consistent with this, we observed no effect of Rac1 inhibitory peptide on TCR/CD28-induced T-cell IL-2 production *ex vivo*. However, we did note that Rac1 inhibition decreased the ability of T cells to produce the inflammatory cytokines TNFα, IFNγ, and IL-17 in response to TCR/CD28 triggering. These findings bear similarities with a recent report that thionamides interfere with human T-cell TNFα and IFNγ production via inhibition of Rac1 activation [41]. Interference with anti-bCII production in CIA might also be attributable to direct suppression of TCR/CD28 signaling as we observe that ICOS and CD154 expression is compromised in T cells treated with Rac1 peptide. Sequential upregulation of these two proteins plays a requisite role in providing costimulatory signals required for efficient B-cell antibody production and isotype class switching [42,43]. Blockade of either ICOS or CD154 interaction with their respective ligands is protective in murine models of arthritis and suppresses autoantibody production [33-36]. In RA, synovial T-cell expression of CD154 can also promote antigen-independent activation of macrophages and stromal cells [44-46]. Finally, Rac1 inhibition may interfere with anti-bCII antibody production via effects on DCs. Although this possibility is not addressed in our studies, Rac1-deficient CD8α^+ ^DCs, but not Rac2-deficient DCs, fail to migrate to secondary lymphoid organs and cannot establish stable contacts with naïve T cells [22]. DCs from mindin^-/- ^mice, which have reduced expression of Rac1 and Rac2, also have impaired priming capacity due to inefficient engagement with T cells, in turn leading to defective humoral responses to T cell-dependent antigens in these mice [47].

Our studies suggest that Rac1 peptide suppresses T-cell activation and subsequent generation of inflammatory cytokines and costimulatory proteins needed for anti-collagen antibody production in CIA. Future studies will be required not only to further define specific effects on lymphocytes and lymphocyte interactions with APCs but also to consider the potential contributions of Rac2, independently or in conjunction with Rac1, to pathology in CIA. Structure-based studies have recently led to the development of small-molecular-weight compounds that can specifically prevent interaction of Rac1 and Rac2 with activating guanine nucleotide exchange factors [27,48]. These compounds can block RA FLS growth and matrix invasion *in vitro*, although their efficacy in the treatment of arthritis *in vivo *remains to be established.

## Conclusions

We demonstrate that a cell-permeable inhibitory Rac1 carboxy-terminal peptide can reduce paw swelling and antibody production during murine experimental arthritis. These protective effects may be attributed at least in part to suppression of T-cell inflammatory cytokine and costimulatory protein expression. Conceivably, Rac1 peptide treatment could augment the pharmacological activity toward B-lineage cells of other immunosuppressive therapies, like rituximab or atacicept, which may theoretically increase therapeutic activity. An alternative approach that might perhaps result in a beneficial effect on both clinical signs and symptoms as well as joint destruction could be the suppression of Rac signaling in RA by compounds targeting both Rac1 and Rac2 signaling. The present study supports the rationale for future studies exploring these approaches.

## Abbreviations

APC: antigen-presenting cell; bCII: bovine collagen type II; CFA: complete Freund's adjuvant; CIA: collagen-induced arthritis; Ctrl: control; DC: dendritic cell; EDTA: ethylenediaminetetraacetic acid; FACS: fluorescence-activated cell sorting; FITC: fluorescein isothiocyanate; FLS: fibroblast-like synoviocyte; IFNγ: interferon-gamma; Ig: immunoglobulin; IL: interleukin; MFI: mean fluorescence intensity; PBS: phosphate-buffered saline; PE: phycoerythrin; RA: rheumatoid arthritis; SDF-1α: stromal cell-derived factor 1 alpha; TCR: T-cell receptor; TNFα: tumor necrosis factor-alpha.

## Competing interests

The authors declare that they have no competing interests.

## Authors' contributions

JRFA performed and evaluated the animal experiments, conducted statistical analyses, and drafted the manuscript. WD and SK performed and evaluated animal experiments and T-cell phenotyping and activation experiments. DdL assisted in the animal experiments. PBvH and A-MvS performed the actin polymerization experiments. J-PtK generated the cell-permeable peptides. MES performed the enzyme-linked immunosorbent assays. KAR coordinated the study, evaluated data, and drafted the manuscript. MJV, PLH, and PPT conceived the study and its design. All authors read and approved the final manuscript.
